# Supplementary material for: Whole genome analysis for 163 gRNAs in Cas9-edited mice reveals minimal off-target activity
Source: Commun Biol. 2023 Jun 10;6:626. doi: 10.1038/s42003-023-04974-0 (PMC10257658; doi:10.1038/s42003-023-04974-0)
Supplement: Supplementary file 2 — Supplementary Information [file 42003_2023_4974_MOESM2_ESM.pdf]

Whole genome analysis for 163 guide RNAs in Cas9-edited mice reveals minimal off-target activity

Kevin A. Peterson, Sam Khalouei, Nour Hanafi, Joshua A. Wood, Denise G. Lanza, Lauri G. Lintott, Brandon Willis, John R. Seavitt, Robert E. Braun, Mary E. Dickinson, Jacqueline K. White, K.C. Kent Lloyd, Jason D. Heaney, Stephen A. Murray, Arun Ramani, and Lauryl M.J. Nutter*

*Corresponding author: lauryl.nutter@sickkids.ca

Supplementary Information

Supplementary Data 1. Sample and guide RNA information for whole genome sequence analysis.

Supplementary Data 2. Number of variants removed for each sample with each filtering step.

Supplementary Data 3. Variant calls for off-target sites detected in sequence analysis pipeline.

Supplementary Data 4. Intersection of target guide sites and variants identified in control samples.

Supplementary Data 5. Source data for Fig. 2 and Fig. 3b.

Supplementary Data 6. Provides methods details specific to each participating research centre.

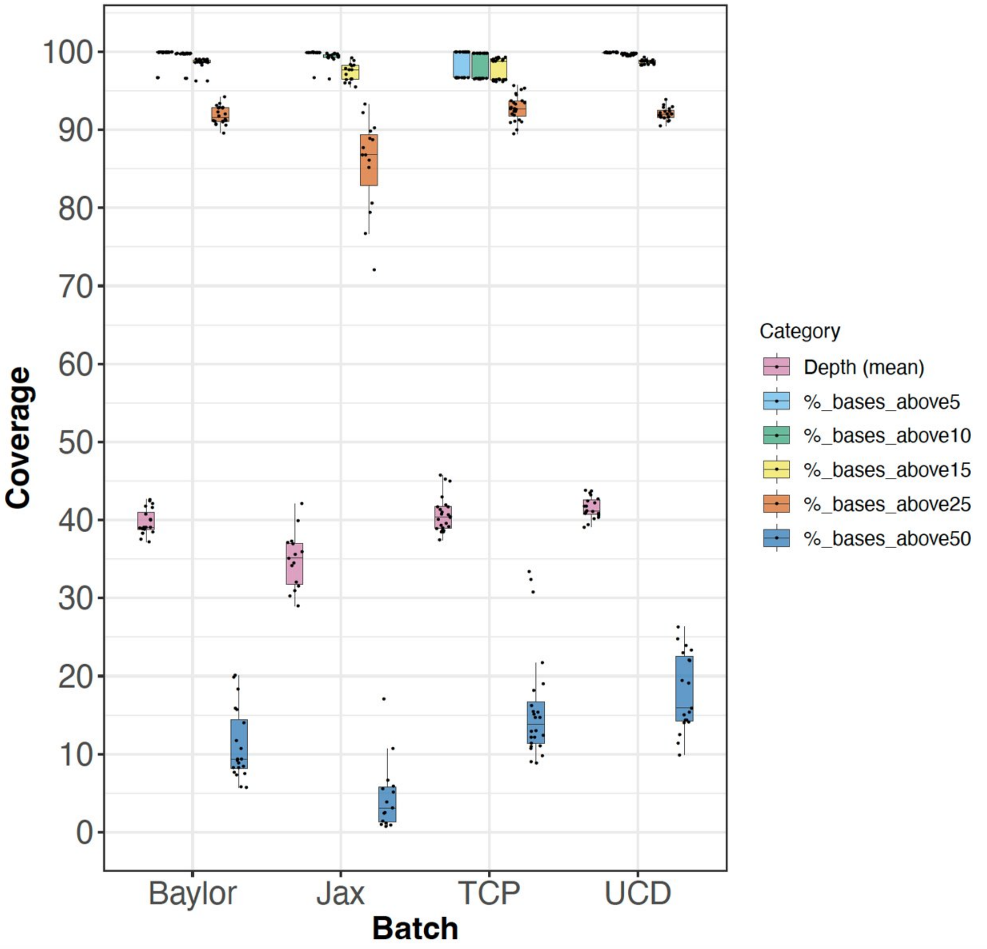

Supplementary Figure 1. Summary of whole-genome sequencing data obtained from each KOMP2 production center showing base pair quality and depth. Lines within the boxes represent medians, lower edges the 1st quartile, and upper edges the 3rd quartile. The length of the box represents the interquartile range (IQR). Whiskers extend to the minimum or maximum values that fall within 1.5 X IQR of the 1st or 3rd quartiles, respectively. Each datapoint represents an individual sample, with the sample size per group as follows: n = 20 Baylor mice, n = 15 Jax mice, n = 24 TCP mice, and n = 19 UCD mice, all biologically independent.

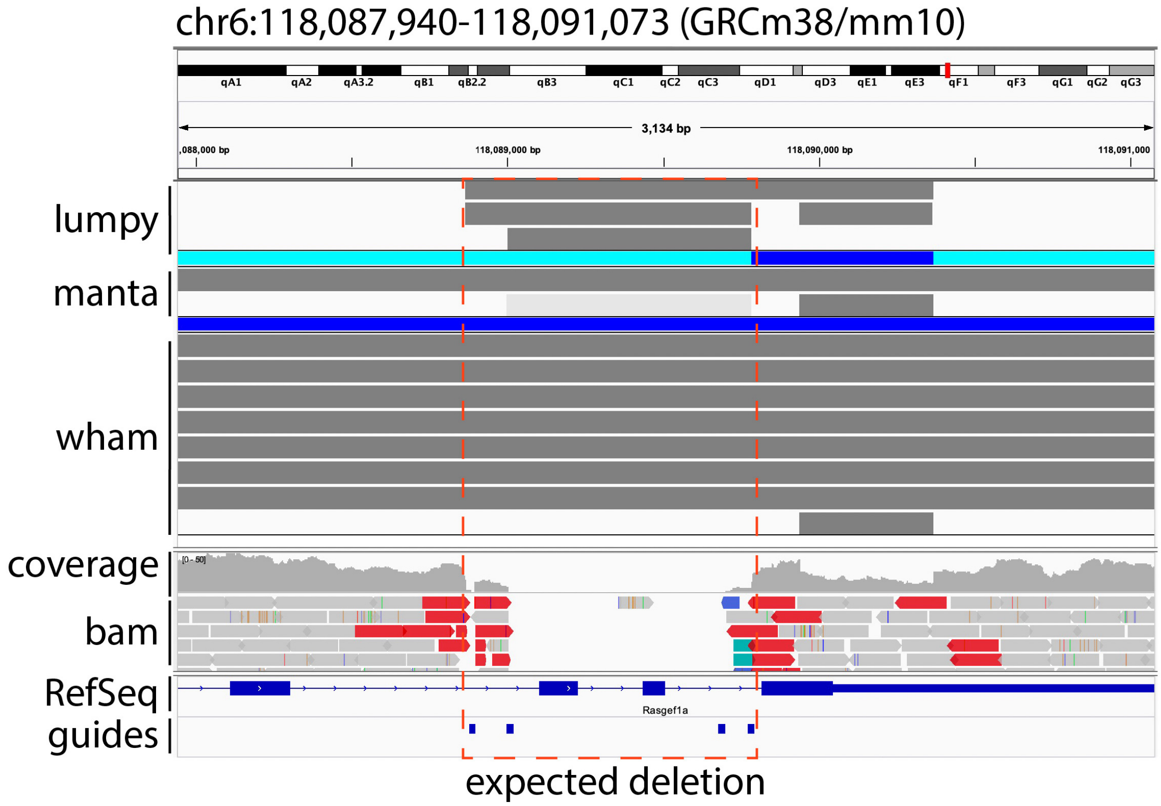

Supplementary Figure 2. Comparison of structural variant callers for *Rasgef1a* deletion. The expected region is depicted with a red box and shows reduced sequence coverage consistent with Cas9 mediated deletion using a four-guide design strategy. The deletion was successfully called by lumpy and manta but filtered out by manta due to low confidence genotype filter indicted by light gray bar. This filtering resulted in the *Rasgef1a* deletion failing to meet the minimum threshold of being independently identified by at least two programs.

a

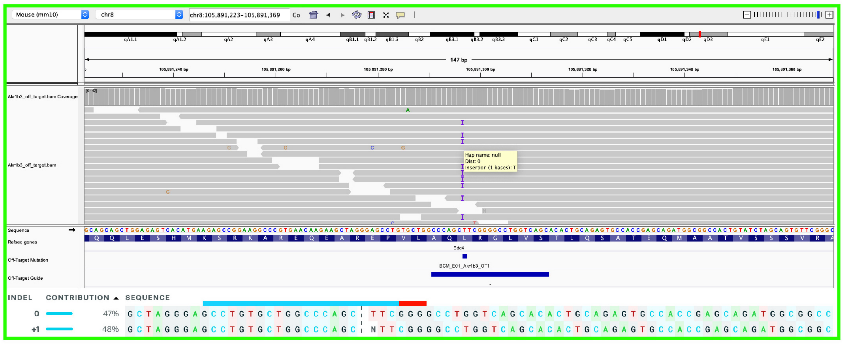

b

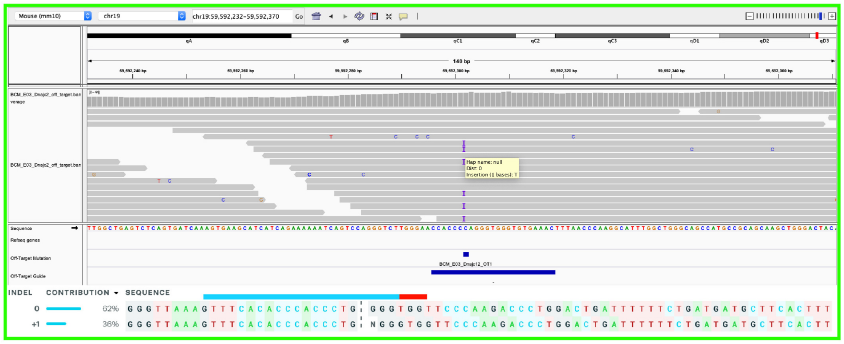

c

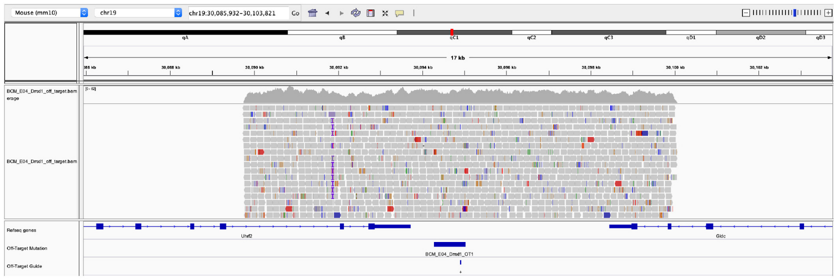

Supplementary Figure 3. Whole-genome sequencing evidence for detected Cas9 off-target activity for sites associated with guides containing 5 or fewer mismatches. (**a-c**) IGV screen shots for each region showing coverage, read data, refSeq gene, computationally detected off-target site and position of off-target guide with +/- strand indicated below relative to PAM sequence. Events confirmed by Sanger sequencing contain a green box with ICE results displayed below indicating the detected off-target mutation and estimated percentage of allele contribution. A light blue bar is drawn above the guide sequence and a red bar above the PAM with a vertical dash line indicating Cas9 cut site at the -3 position relative to PAM.

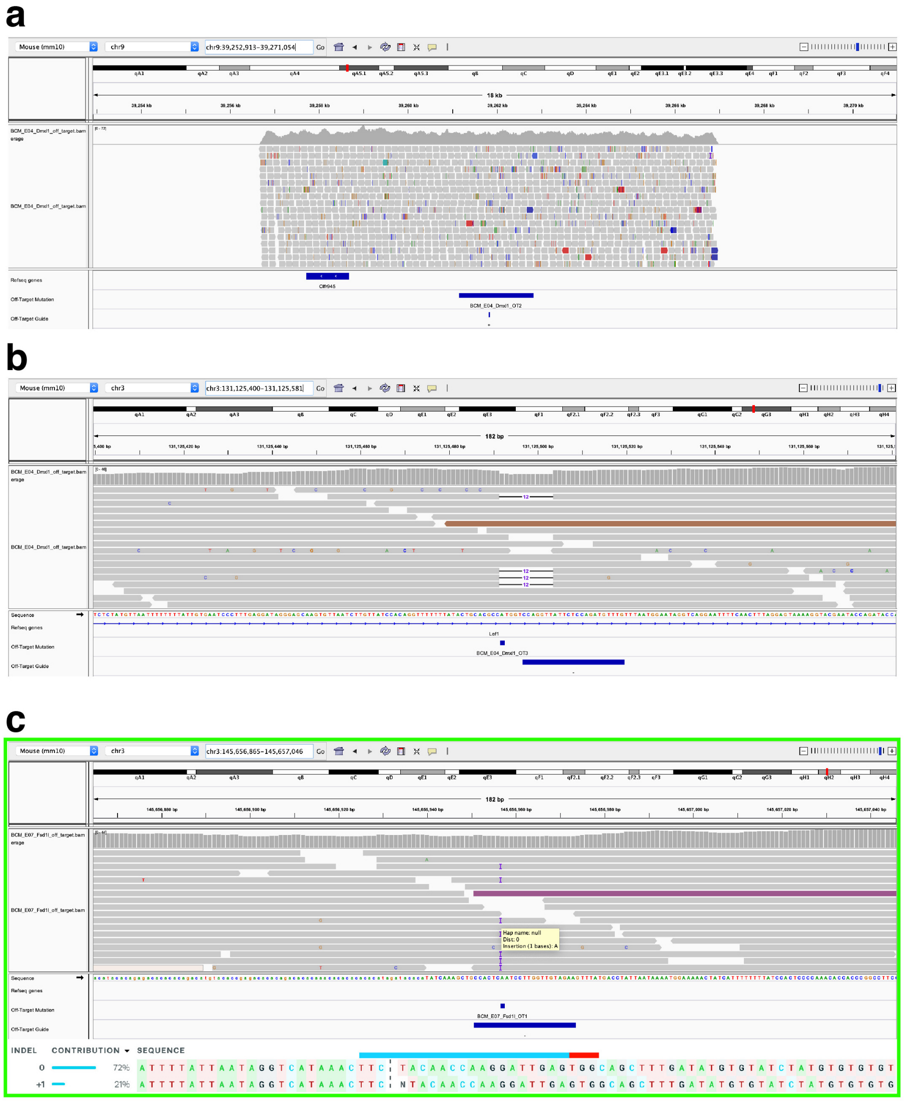

Supplementary Figure 4. Whole-genome sequencing evidence for detected Cas9 off-target activity for sites associated with guides containing 5 or fewer mismatches. (**a-c**) IGV screen shots for each region showing coverage, read data, refSeq gene, computationally detected off-target site and position of off-target guide with +/- strand indicated below relative to PAM sequence. Events confirmed by Sanger sequencing contain a green box with ICE results displayed below indicating the detected off-target mutation and estimated percentage of allele contribution. A light blue bar is drawn above the guide sequence and a red bar above the PAM with a vertical dash line indicating Cas9 cut site at the -3 position relative to PAM.

a

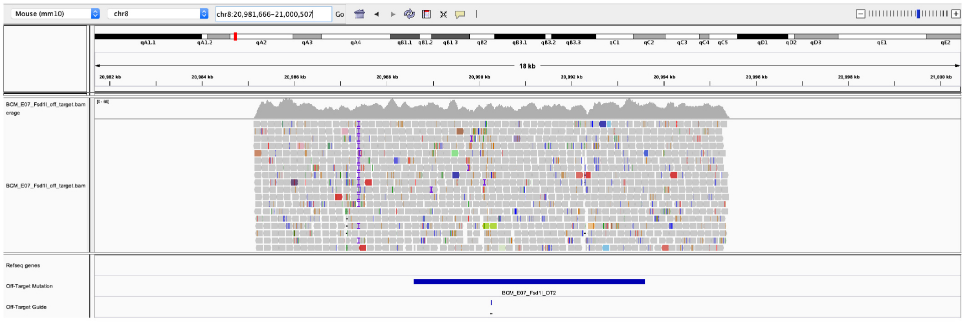

b

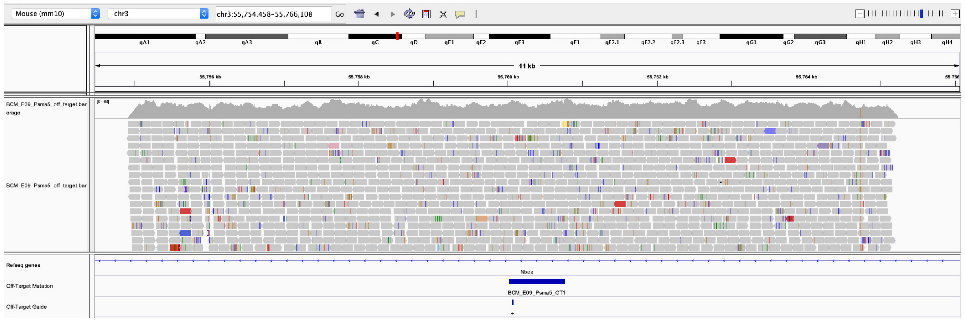

c

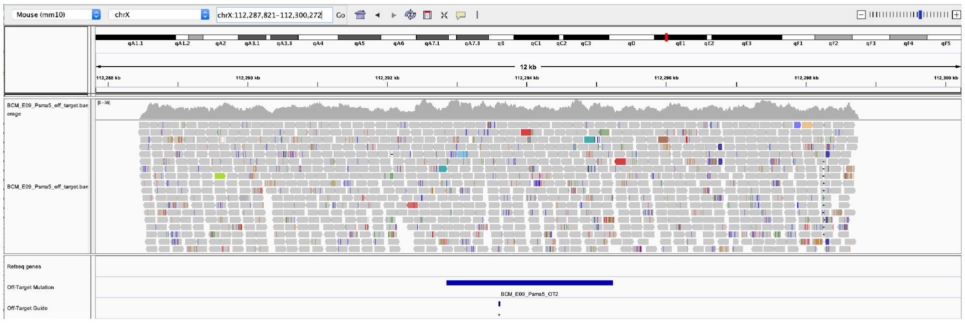

Supplementary Figure 5. Whole-genome sequencing evidence for detected Cas9 off-target activity for sites associated with guides containing 5 or fewer mismatches. (**a-c**) IGV screen shots for each region showing coverage, read data, refSeq gene, computationally detected off-target site and position of off-target guide with +/- strand indicated below relative to PAM sequence.

a

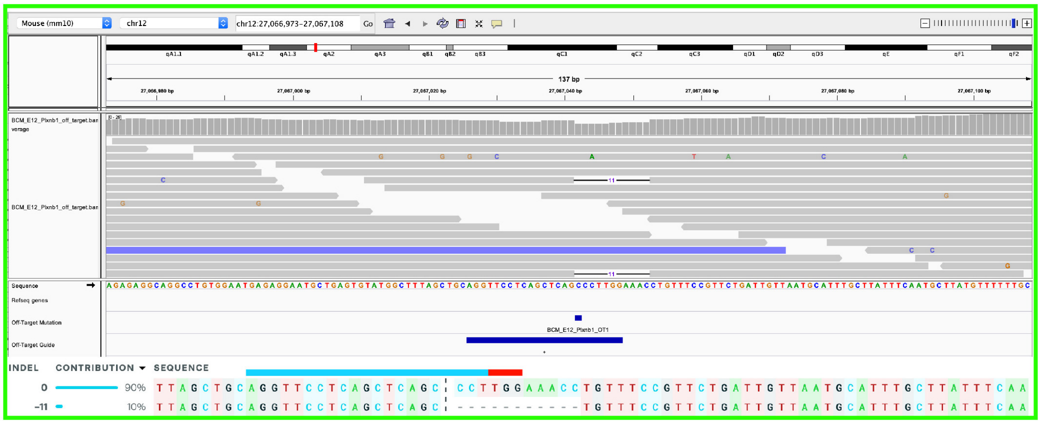

b

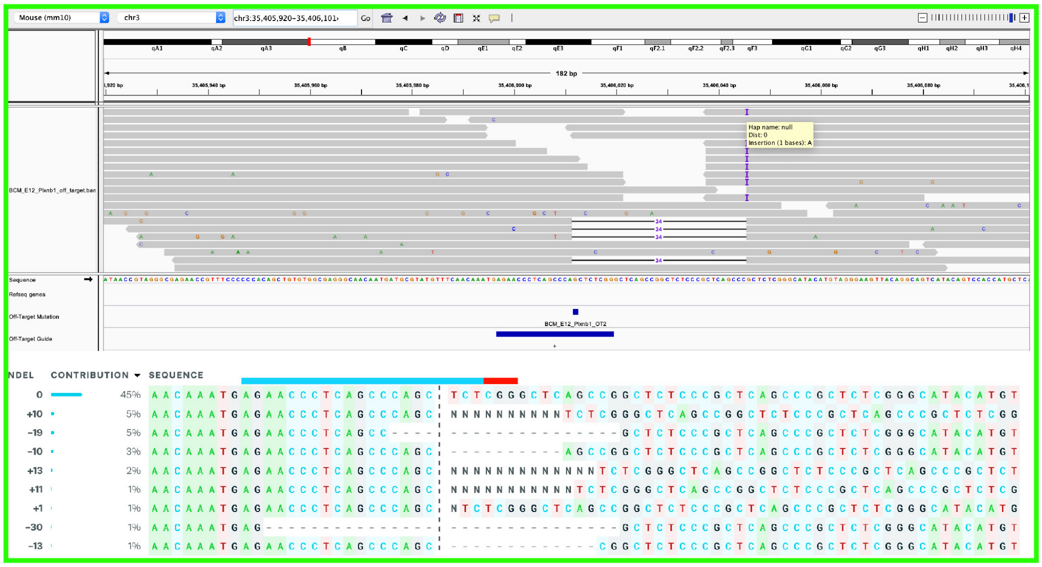

Supplementary Figure 6. Whole-genome sequencing evidence for detected Cas9 off-target activity for sites associated with guides containing 5 or fewer mismatches. (**a-b**) IGV screen shots for each region showing coverage, read data, refSeq gene, computationally detected off-target site and position of off-target guide with +/- strand indicated below relative to PAM sequence. Events confirmed by Sanger sequencing contain a green box with ICE results displayed below indicating the detected off-target mutation and estimated percentage of allele contribution. A light blue bar is drawn above the guide sequence and a red bar above the PAM with a vertical dash line indicating Cas9 cut site at the -3 position relative to PAM.

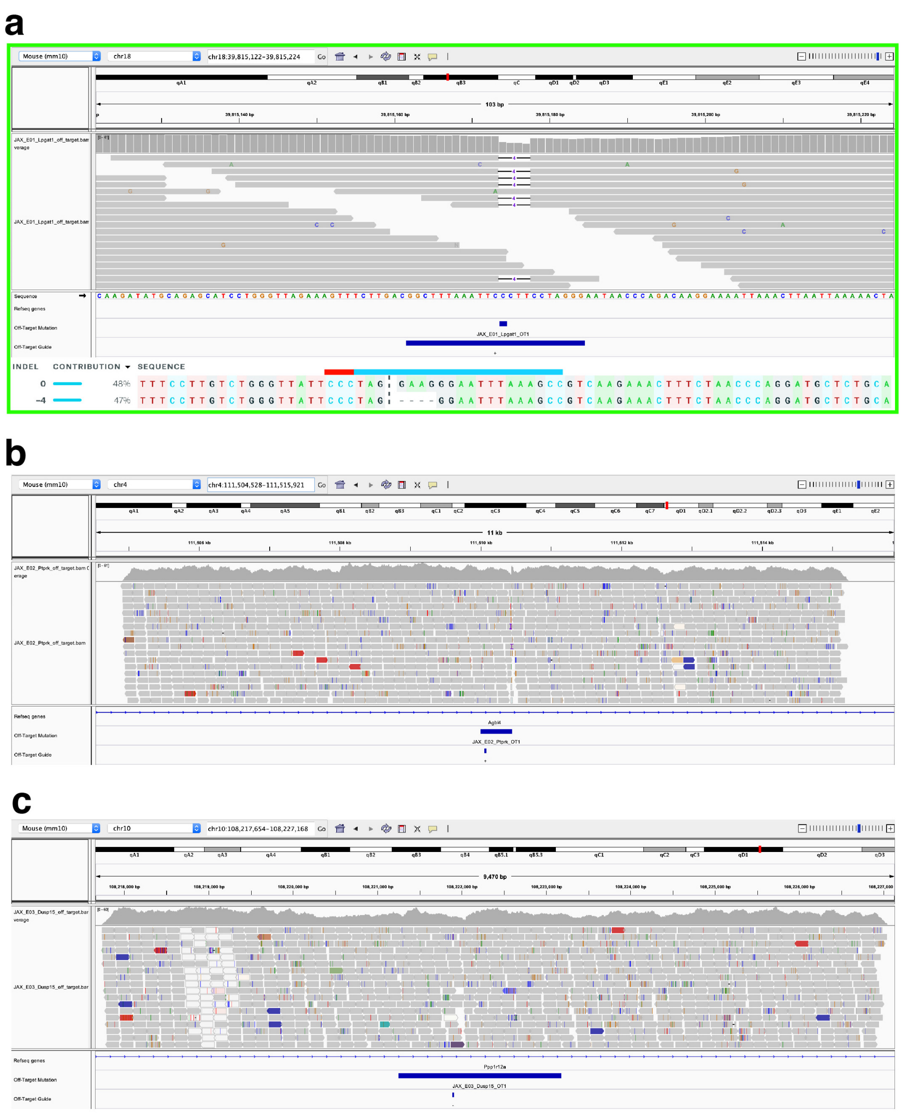

Supplementary Figure 7. Whole-genome sequencing evidence for detected Cas9 off-target activity for sites associated with guides containing 5 or fewer mismatches. (**a-c**) IGV screen shots for each region showing coverage, read data, refSeq gene, computationally detected off-target site and position of off-target guide with +/- strand indicated below relative to PAM sequence. Events confirmed by Sanger sequencing contain a green box with ICE results displayed below indicating the detected off-target mutation and estimated percentage of allele contribution. A light blue bar is drawn above the guide sequence and a red bar above the PAM with a vertical dash line indicating Cas9 cut site at the -3 position relative to PAM.

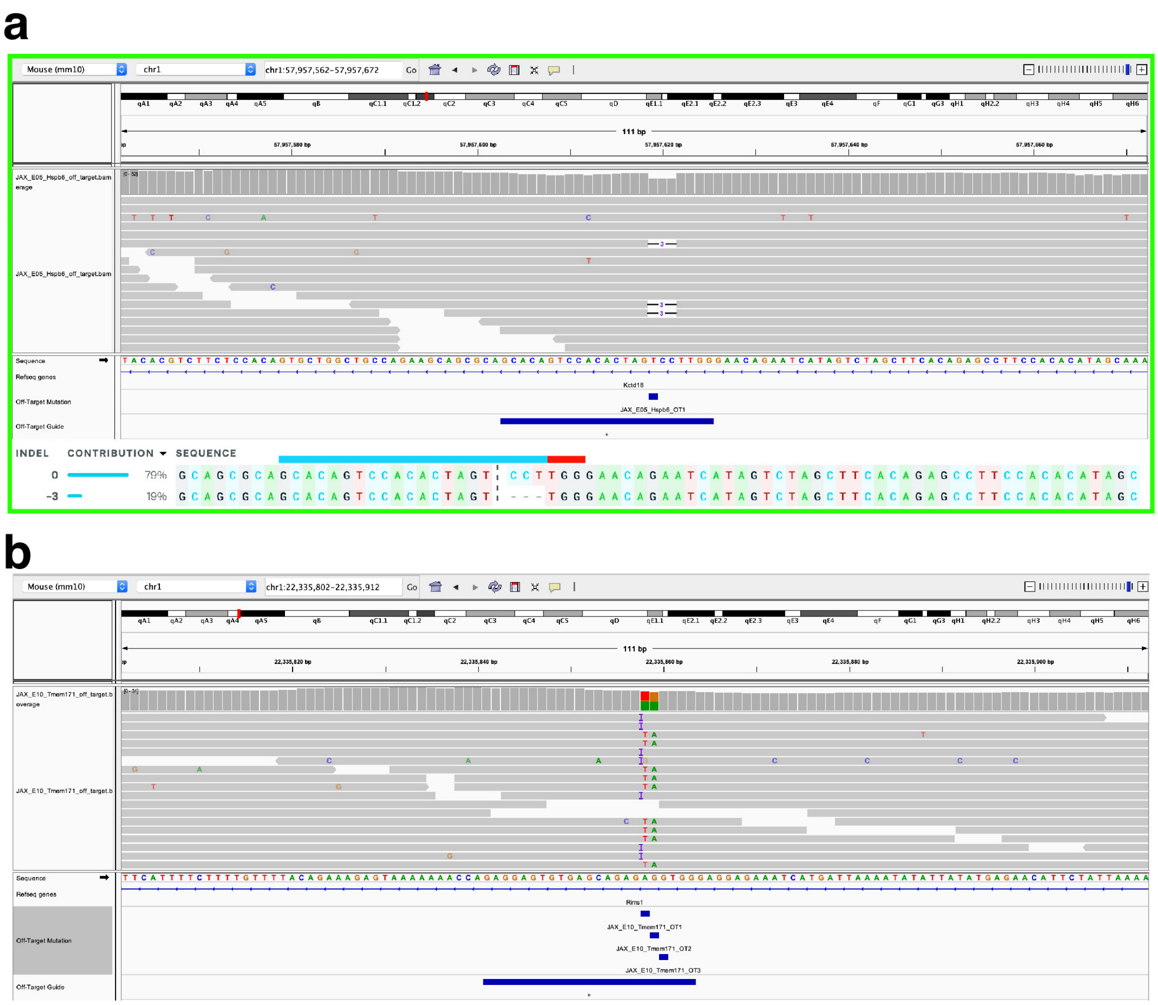

Supplementary Figure 8. Whole-genome sequencing evidence for detected Cas9 off-target activity for sites associated with guides containing 5 or fewer mismatches. (**a-b**) IGV screen shots for each region showing coverage, read data, refSeq gene, computationally detected off-target site and position of off-target guide with +/- strand indicated below relative to PAM sequence. Events confirmed by Sanger sequencing contain a green box with ICE results displayed below indicating the detected off-target mutation and estimated percentage of allele contribution. A light blue bar is drawn above the guide sequence and a red bar above the PAM with a vertical dash line indicating Cas9 cut site at the -3 position relative to PAM.

a

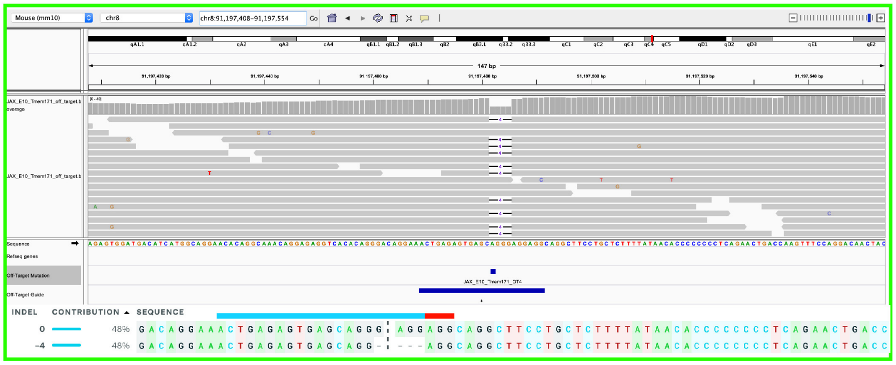

b

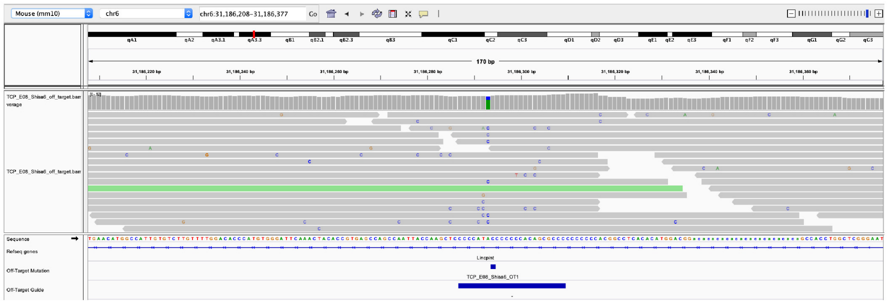

c

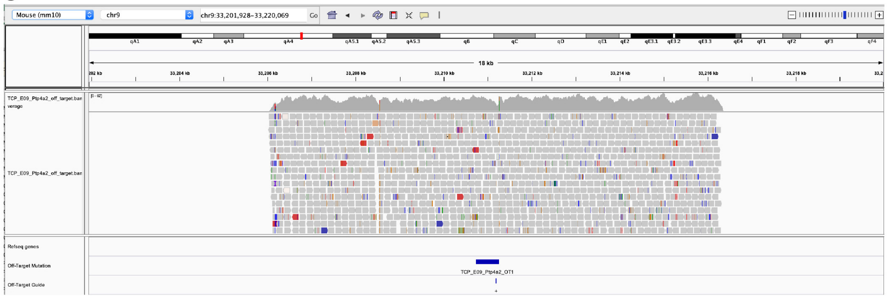

Supplementary Figure 9. Whole-genome sequencing evidence for detected Cas9 off-target activity for sites associated with guides containing 5 or fewer mismatches. (**a-c**) IGV screen shots for each region showing coverage, read data, refSeq gene, computationally detected off-target site and position of off-target guide with +/- strand indicated below relative to PAM sequence. Events confirmed by Sanger sequencing contain a green box with ICE results displayed below indicating the detected off-target mutation and estimated percentage of allele contribution. A light blue bar is drawn above the guide sequence and a red bar above the PAM with a vertical dash line indicating Cas9 cut site at the -3 position relative to PAM.

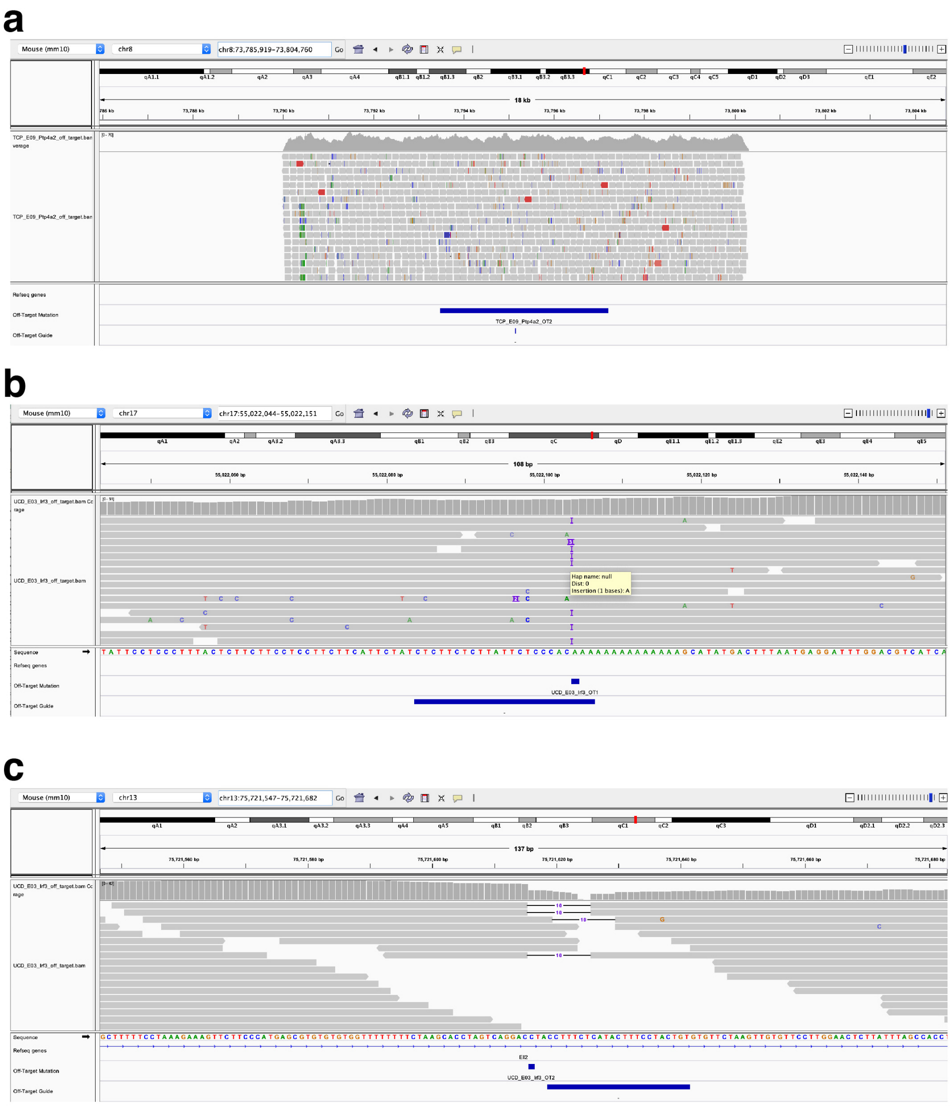

Supplementary Figure 10. Whole-genome sequencing evidence for detected Cas9 off-target activity for sites associated with guides containing 5 or fewer mismatches. (**a-c**) IGV screen shots for each region showing coverage, read data, refSeq gene, computationally detected off-target site and position of off-target guide with +/- strand indicated below relative to PAM sequence. Note, off-target mutationn shown in panel c was confirmed via CIRCLE-seq.

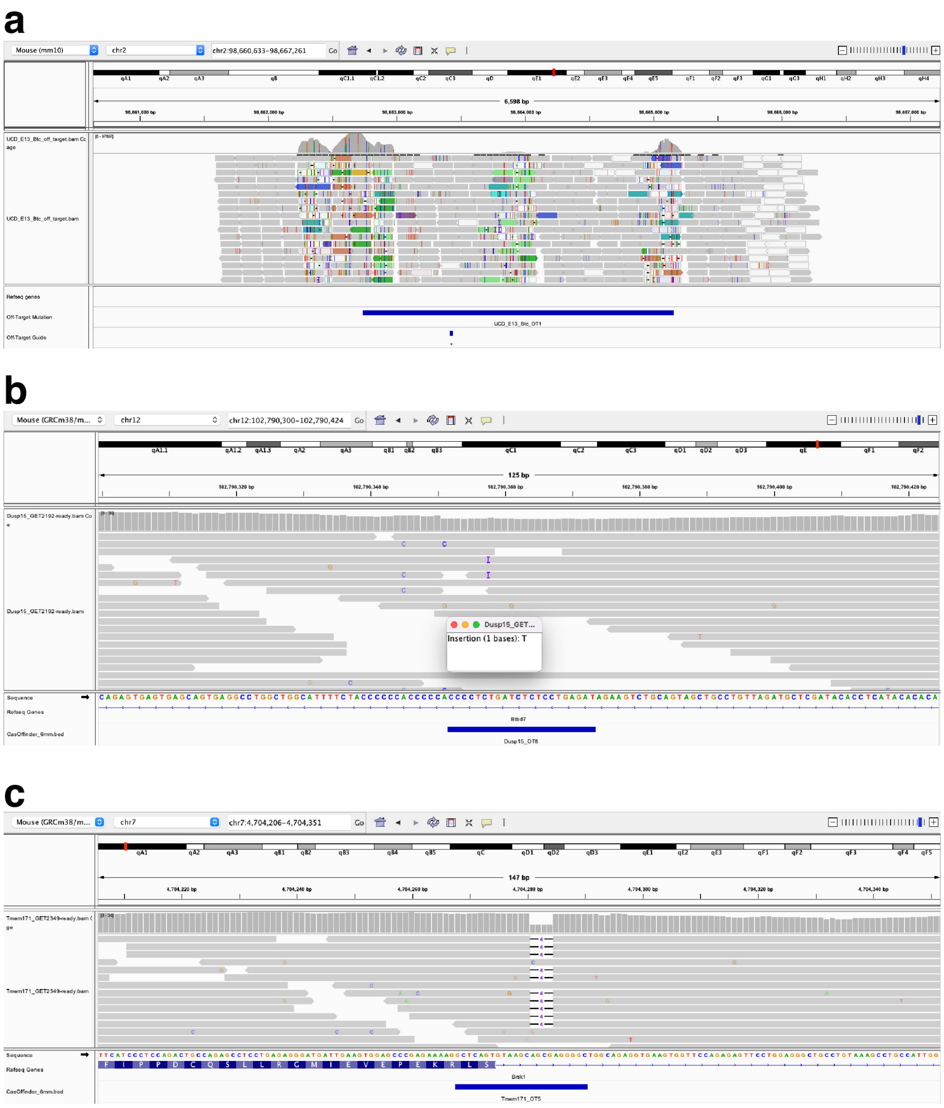

Supplementary Figure 11. Whole-genome sequencing evidence for detected Cas9 off-target activity for sites associated with guides containing 5 or fewer mismatches. (**a-c**) IGV screen shots for each region showing coverage, read data, refSeq gene, computationally detected off-target site and position of off-target guide with +/- strand indicated below relative to PAM sequence. Note, off-target mutations shown in panels b,c were identified via CIRCLE-seq.

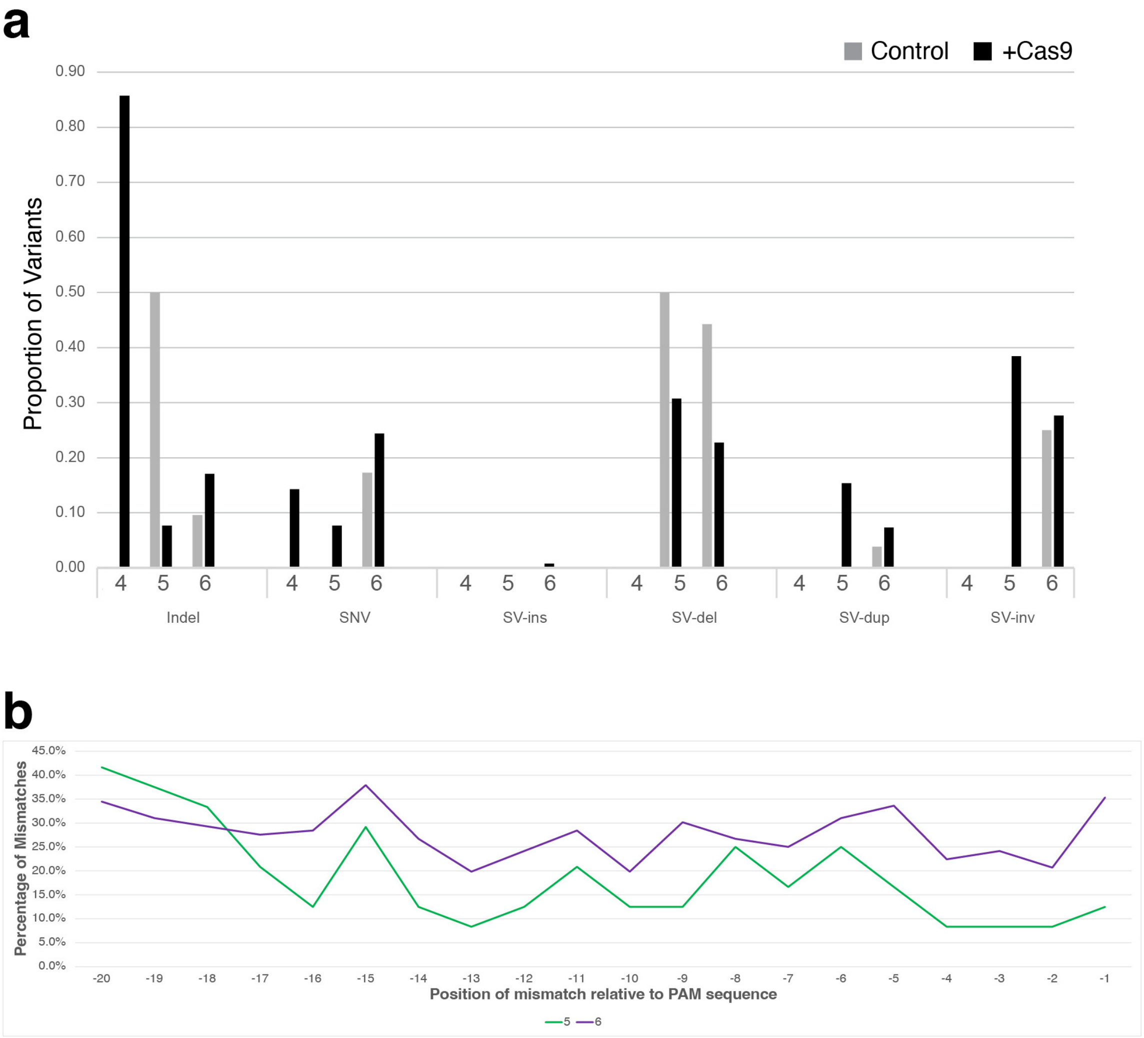

Supplementary Figure 12. Comparison of predicted off-target mismatch allowance, variant class and positional dependence. (**a**) The proportion of variants overlapping a predicted off-target guide site in control animals compared to Cas9 gene-edited (+Cas9). All of the variants detected in control samples were associated with guides with 5 or 6 mismatches. (**b**) Percentage of mismatches identified in off-target guides allowing up to 5 or 6 mismatches and one DNA/RNA bulge. Positions are numbered with -1 adjacent to NRG PAM sequence and -20 most distal.
