# Supplementary material for: Whole genome analysis for 163 gRNAs in Cas9-edited mice reveals minimal off-target activity
Source: Commun Biol. 2023 Jun 10;6:626. doi: 10.1038/s42003-023-04974-0 (PMC10257658; doi:10.1038/s42003-023-04974-0)
Supplement: Supplementary file 3 — Description of Additional Supplementary Files [file 42003_2023_4974_MOESM3_ESM.pdf]

Description of Additional Supplementary Files

File Name: Supplementary Data 1

Description: Sample and gRNA information used for whole-genome sequencing, gene editing, and off-target site prediction.

File Name: Supplementary Data 2

Description: Number of variants remaining for each sample after each filtering step and number of controls that shared a given variant in each sample and filtered out by our secondary filter step.

File Name: Supplementary Data 3

Description: Detailed information about each off-target site detected in the WGS data of Cas9-edited mice in our pipeline or by CIRCLE-Seq.

File Name: Supplementary Data 4

Description: Detailed information about each predicted off-target site detected in WGS data of control mice.

File Name: Supplementary Data 5

Description: Source data for Figures 2 and 3b.

File Name: Supplementary Data 6

Description: Provides additional information for the Methods.
