# Supplementary material for: Whole genome analysis for 163 gRNAs in Cas9-edited mice reveals minimal off-target activity
Source: Commun Biol. 2023 Jun 10;6:626. doi: 10.1038/s42003-023-04974-0 (PMC10257658; doi:10.1038/s42003-023-04974-0)
Supplement: Supplementary file 10 — Reporting Summary [file 42003_2023_4974_MOESM10_ESM.pdf]

nature portfolio

Corresponding author(s): Nutter, LMJ
Last updated by author(s): Apr 29, 2023

Reporting Summary

Nature Portfolio wishes to improve the reproducibility of the work that we publish. This form provides structure for consistency and transparency in reporting. For further information on Nature Portfolio policies, see our Editorial Policies and the Editorial Policy Checklist.

Statistics

For all statistical analyses, confirm that the following items are present in the figure legend, table legend, main text, or Methods section.

n/a	Confirmed	
☐	☒	The exact sample size (*n*) for each experimental group/condition, given as a discrete number and unit of measurement
☐	☒	A statement on whether measurements were taken from distinct samples or whether the same sample was measured repeatedly
☐	☒	The statistical test(s) used AND whether they are one- or two-sided<br>*Only common tests should be described solely by name; describe more complex techniques in the Methods section.*
☐	☒	A description of all covariates tested
☐	☒	A description of any assumptions or corrections, such as tests of normality and adjustment for multiple comparisons
☐	☒	A full description of the statistical parameters including central tendency (e.g. means) or other basic estimates (e.g. regression coefficient) AND variation (e.g. standard deviation) or associated estimates of uncertainty (e.g. confidence intervals)
☐	☒	For null hypothesis testing, the test statistic (e.g. *F*, *t*, *r*) with confidence intervals, effect sizes, degrees of freedom and *P* value noted<br>*Give P values as exact values whenever suitable.*
☒	☐	For Bayesian analysis, information on the choice of priors and Markov chain Monte Carlo settings
☒	☐	For hierarchical and complex designs, identification of the appropriate level for tests and full reporting of outcomes
☒	☐	Estimates of effect sizes (e.g. Cohen's *d*, Pearson's *r*), indicating how they were calculated

Our web collection on statistics for biologists contains articles on many of the points above.

Software and code

Policy information about availability of computer code

Data collection	Whole genome sequencing data was collected using the software associated with the HiSeqX used to do the sequencing.
Data analysis	As described in detail in the methods section: We used FastqC (http://www.bioinformatics.babraham.ac.uk/projects/fastqc/) and fastQ Screen32 to assess sequence quality. Reads were then processed through the bcbio pipeline (https://github.com/bcbio/bcbio-nextgen, v1.1.0) for all steps from alignment to variant calling with the following: alignment to GRCm38 with bwa-mem; GATK 4.0 to call variants; resulting VCF files filtered with bcftoos (v1.6); custom python script to remove alleles with frequencies <0.2; callable intervals defined custom script (github??) and bedtools multiinter (v2.27.1); removing shared variants with bcbio-variation-recall (v0.1.7) and bcftools isec. Structural variants were called by lumpy, manta, CNVkit, and Wham followed by Metasv. SnpEff (v4.3t) was used to map intergenic, exonic and intronic variants. To compare means between groups "compare_means" of R package ggpubr (https://rpkgs.datanovia.com/ggpubr/) was used which provided the Wilcoxon test followed by Bonferroni correction. Predicted off-targets were identified using Cas-OFFinder21 and visually assessed using IGV (Integrated Genome Viewer). Heatmap dendrogram was created by providing the percentage of common variants between any two samples (using “bcftools isec” tool and custom scripts) as input to the pheatmap R package (https://cran.r-project.org/web/packages/pheatmap/). Custom data parsing scripts are available at https://github.com/The-Centre-for-Phenogenomics/Cas9-WGS and Zenodo at https://doi.org/10.5281/zenodo.7823655.

For manuscripts utilizing custom algorithms or software that are central to the research but not yet described in published literature, software must be made available to editors and reviewers. We strongly encourage code deposition in a community repository (e.g. GitHub). See the Nature Portfolio guidelines for submitting code & software for further information.

Data

Policy information about availability of data

All manuscripts must include a data availability statement. This statement should provide the following information, where applicable:

- Accession codes, unique identifiers, or web links for publicly available datasets
- A description of any restrictions on data availability
- For clinical datasets or third party data, please ensure that the statement adheres to our policy

Sequence data associated with this study are deposited to the NCBI Sequence Read Archive (SRA) under accession number PRJNA687003.

Research involving human participants, their data, or biological material

Policy information about studies with human participants or human data. See also policy information about sex, gender (identity/presentation), and sexual orientation and race, ethnicity and racism.

Reporting on sex and gender	not applicable
Reporting on race, ethnicity, or other socially relevant groupings	not applicable
Population characteristics	not applicable
Recruitment	not applicable
Ethics oversight	not applicable

Note that full information on the approval of the study protocol must also be provided in the manuscript.

Field-specific reporting

Please select the one below that is the best fit for your research. If you are not sure, read the appropriate sections before making your selection.

☒ Life sciences ☐ Behavioural & social sciences ☐ Ecological, evolutionary & environmental sciences

For a reference copy of the document with all sections, see nature.com/documents/nr-reporting-summary-flat.pdf

Life sciences study design

All studies must disclose on these points even when the disclosure is negative.

Sample size	No pre-calculation of sample sizes was done. This study analyzes whole genome sequence data from founder mice generated using 2 to 4 gRNAs per mouse for a total of 163 guides in 78 founder mice from 4 different centres. Between 5 and 12 control mice were analyzed at each centre. To our knowledge, this sample size is the largest representation of gRNAs used to assess in vivo off-target Cas9 activity. Variation between control and experimental data sets was similar, so we concluded that the number of controls was sufficient for the size of the experimental data set.
Data exclusions	No data was excluded from the study. However, due to a technical issue, one sample was omitted from complete SV analysis. This is described in the manuscript "All samples successfully passed the MetaSV step, with the exception of the Nat8 sample, which encountered an unresolved technical error, and consequently was omitted from the rest of the SV analysis."
Replication	This study used samples from four different laboratories. All data sets provided similar results supporting the conclusions of the study.
Randomization	Embryos from a pool of collected embryos were randomly allocated to groups for treatment with different Cas9-gRNA combinations on each experimental day. When done, unmanipulated embryos or those electroporated without Cas9 were randomly assigned from the same pool of embryos as the manipulated embryos. Control B6 stud males were randomly selected from among the population of stud males used to produce zygotes.
Blinding	Whole genome sequencing was provided as a service at The Centre for Applied Genomics. Technicians there were blinded to sample type. Blinding at analysis was not necessary as the groups needed to be assessed based on how the samples were or were not treated before sequencing.

Reporting for specific materials, systems and methods

We require information from authors about some types of materials, experimental systems and methods used in many studies. Here, indicate whether each material, system or method listed is relevant to your study. If you are not sure if a list item applies to your research, read the appropriate section before selecting a response.

April 2023

Materials & experimental systems

n/a	Involved in the study
x	Antibodies
x	Eukaryotic cell lines
x	Palaeontology and archaeology
	x Animals and other organisms
x	Clinical data
x	Dual use research of concern
x	Plants

Methods

n/a	Involved in the study
x	ChIP-seq
x	Flow cytometry
x	MRI-based neuroimaging

Animals and other research organisms

Policy information about studies involving animals; ARRIVE guidelines recommended for reporting animal research, and Sex and Gender in Research

Laboratory animals	All experiments were performed on C57BL/B6N mice obtained from either The Jackson Laboratory (C57BL/6NJ; stock #5304) or Charles River (C57BL/6NCrl; strain code 027). Animals were 16-32 weeks of age when euthanized for sample collection. The specific strain and sex of each animal used is listed in Supplementary Data 1.
Wild animals	Not applicable.
Reporting on sex	The study included both male and female animals and findings apply to both sexes. Cas9-mediated editing and off-target activity occurs in early embryos before sex determination. As a result, analysis by sex was not performed. Experimental animals were selected based on whether they had and transmitted to the next generation the desired genome edit. Due to the ability to generate more progeny from a male than female mouse, male mice were often selected, when available, for germline transmission test breeding. Control DNA from stud males was necessarily obtained from male mice. DNA obtained from control embryos was from an equal number of male and female mice. In total 64 male DNA samples and 14 female DNA samples were used in this study.
Field-collected samples	Not applicable.
Ethics oversight	All procedures involving animals at The Centre for Phenogenomics (TCP) were performed in compliance with the Animals for Research Act of Ontario and the Guidelines of the Canadian Council on Animal Care under Animal Use Protocols 0008, 0084 and 0275 reviewed and approved by the TCP's Animal Care Committee. All animal use at Baylor College of Medicine, The Jackson Laboratory and UC Davis were done in accordance with the Animal Welfare Act and the AVMA Guidelines on Euthanasia, in compliance with the ILAR Guide for Care and Use of Laboratory Animals, and with prior approval from their respective institutional animal care and use committees (IACUC).

Note that full information on the approval of the study protocol must also be provided in the manuscript.
